# Factors influencing participation in a randomized controlled resistance exercise intervention study in breast cancer patients during radiotherapy

**DOI:** 10.1186/s12885-015-1213-1

**Published:** 2015-03-27

**Authors:** Sandra M Gollhofer, Joachim Wiskemann, Martina E Schmidt, Oliver Klassen, Cornelia M Ulrich, Jan Oelmann, Holger Hof, Karin Potthoff, Karen Steindorf

**Affiliations:** Unit of Physical Activity and Cancer, Department of Preventive Oncology, German Cancer Research Center and National Center for Tumor Diseases, Im Neuenheimer Feld 460, 69120 Heidelberg, Germany; Unit of Environmental Epidemiology, German Cancer Research Center, Im Neuenheimer Feld 280, 69120 Heidelberg, Germany; Department of Medical Oncology, National Center for Tumor Diseases, Im Neuenheimer Feld 460, 69120 Heidelberg, Germany; Department of Radiation Oncology, University of Heidelberg Medical Center, Im Neuenheimer Feld 400, 69120 Heidelberg, Germany; Department of Preventive Oncology, National Center for Tumor Diseases, Im Neuenheimer Feld 460, 69120 Heidelberg, Germany; National Center for Tumor Diseases (NCT), Divisions of Medical Oncology and Preventive Oncology, Im Neuenheimer Feld 460, 69120 Heidelberg, Germany

**Keywords:** Clinical trial, Physical activity, Oncology, Randomized controlled trial, Recruitment

## Abstract

**Background:**

Over the past years knowledge about benefits of physical activity after cancer is evolving from randomized exercise intervention trials. However, it has been argued that results may be biased by selective participation. Therefore, we investigated factors influencing participation in a randomized exercise intervention trial for breast cancer patients.

**Methods:**

Non-metastatic breast cancer patients were systematically screened for a randomized exercise intervention trial on cancer-related fatigue. Participants and nonparticipants were compared concerning sociodemographic characteristics (age, marital status, living status, travel time to the training facility), clinical data (body-mass-index, tumor stage, tumor size and lymph node status, comorbidities, chemotherapy), fatigue, and physical activity. Reasons for participation or declination were recorded.

**Results:**

117 patients (52 participants, 65 nonparticipants) were evaluable for analysis. Multiple regression analyses revealed significantly higher odds to decline participation among patients with longer travel time (p = 0.0012), living alone (p = 0.039), with more comorbidities (0.031), previous chemotherapy (p = 0.0066), of age ≥ 70 years (p = 0.025), or being free of fatigue (p = 0.0007). No associations were found with BMI or physical activity. By far the most frequently reported reason for declination of participation was too long commuting time to the training facility.

**Conclusions:**

Willingness of breast cancer patients to participate in a randomized exercise intervention study differed by sociodemographic factors and health status. Neither current physical activity level nor BMI appeared to be selective for participation. Reduction of personal inconveniences and time effort, e.g. by decentralized training facilities or flexible training schedules, seem most promising for enhancing participation in exercise intervention trials.

**Trial registration:**

Registered at ClinicalTrials.gov: NCT01468766 (October 2011).

## Background

In recent years an increasing number of studies have been published, evaluating the role of physical activity during or after cancer treatment. It has been shown that structured exercise training can reduce adverse effects in cancer patients like cancer-related fatigue (CRF) [1-3], improve quality of life [4] and positively impact cardiorespiratory fitness [5]. Most of the findings are based on clinical studies with small numbers of subjects or patients, and there is an ongoing debate whether those patients willing to be randomized to a demanding exercise program might be highly selective [6,7]. Clinical trials are essential for research and randomized controlled studies are considered the gold standard to evaluate new therapies and determine efficacy or effectiveness of interventions [8]. However, only 3-5% of all cancer patients in Great Britain and the US are included in randomized controlled studies [9]. These low rates result from strict study protocols together with patient- and physician related factors, e.g. time constraints or influence on the relationship between physician and patient. Moreover, it has been shown that the process of recruitment itself is very selective and often threatens the generalizability [10]. Data from clinical cancer trials (mostly drug studies) suggest that age [11,12], tumor stage [13,14], lymph node involvement [12], comorbidities [12], and travel distance [15,16] play an important role in the acceptance or non-acceptance in study participation. De Jong et al. [17] determined differences between participants and nonparticipants of an exercise trial for patients with rheumatoid arthritis. They concluded that nonparticipants were older, mainly males, suffered for a longer duration from their disease and had a lower level of education. To our knowledge, the determinants of participation of cancer patients in exercise intervention trials have not yet been systematically investigated in this extent. It is important to explore these determinants to improve participation rates in further trials.

Therefore aim of the present study was to investigate factors that explain participation and nonparticipation in an exercise intervention trial for breast cancer patients undergoing adjuvant radiotherapy. This study focuses on demographic, personal and clinical factors associated with participation, as well as patient motivation.

## Methods

### Study design

The present BEST-Participation-study was carried out from July 2011 to December 2011 during the patient screening for the BEST-study. The BEST-study (in German: **B**ewegung und **E**ntspannung für Brustkrebspatientinnen unter **S**trahlen**t**herapie, translation: exercise and relaxation for breast cancer patients during radiotherapy) is a prospective, randomized, controlled intervention trial for non-metastatic breast cancer patients undergoing adjuvant radiotherapy. The BEST-study compared a 12-week resistance intervention program during and after radiotherapy with relaxation training in regard to cancer-related fatigue perception [18,19]. The BEST-Participation-study was an observational study embedded in the BEST-study, collecting information on factors potentially influencing willingness to participate in a randomized exercise-intervention trial. The aims of the present analysis were (1) to determine clinical and sociodemographic characteristics of participants and nonparticipants, and (2) to identify reasons for participation or declination. Patients, who signed informed consent for the BEST trial automatically took part in the BEST-Participation-study and are referred to as participants throughout this manuscript. Patients, who refused participation in the BEST-study but agreed to fill out the short questionnaire for the BEST-Participation-study, are referred to as nonparticipants.

Protocols of the BEST- and of the BEST-Participation-study were approved by the Ethic Committee of the University of Heidelberg and have been performed in accordance with the ethical standards of the Declaration of Helsinki. The BEST-study is registered at ClinicalTrials.gov (NCT01468766).

### Study population

All participants were treated at the National Center for Tumor Diseases Heidelberg (NCT) in cooperation with the Department of Radiooncology of the University Medical Centre in Heidelberg. Inclusion criteria were: female breast cancer patients with UICC stage I-III, indication for adjuvant radiotherapy, age ≥ 18 years, BMI ≥ 18 kg/m^2^, written informed consent, ability to follow instructions. Exclusion criteria were: acute infection, problems with walking or standing, severe neurological dysfunction, severe cardiovascular disease, severe pulmonary insufficiency, severe renal insufficiency, other malignancies (except carcinoma in situ of skin or cervix), abuse of alcohol or drugs, regular participation in resistance or endurance training. Breast cancer patients were recruited by a physician who was responsible for the assessment of inclusion and exclusion criteria. Both nonparticipants and participants had to fulfill these BEST-inclusion criteria.

### Study recruitment

Eligible patients were briefly informed about the exercise trial at their first consultation for radiotherapy at the outpatient clinic of the Department of Radiooncology of the NCT. Interested patients were handed out an information sheet. They were called a few days later to get more comprehensive information.

Upon informed consent women were included in the BEST-study as well as in the BEST-Participation-study. As participants they had to fill out relevant questionnaires (see below) at their baseline visit for the study entry.

**Figure 1:**
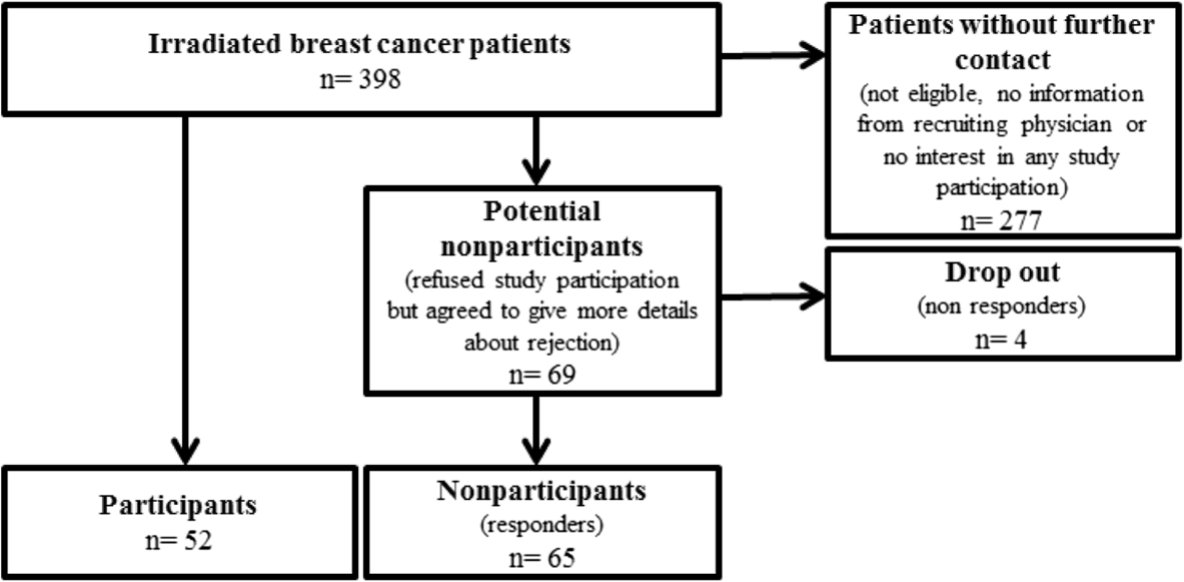
**Recruitment flow.**

Patients refusing participation were asked whether they would be willing to provide some reasons for their declination and to complete a brief questionnaire either immediately in the outpatient clinic or at home, returned by mail. Written informed consent was obtained from both, BEST participants and nonparticipants. For the recruitment flow see Figure 1.

### Data collection

Clinical data, including tumor size, lymph node status, tumor stage, comorbidities, chemotherapy (yes/no), age, BMI, were collected from the clinical information system. Additional information, such as sociodemographic data, fatigue, physical activity and motivational aspects for exercise, was assessed by self-administered questionnaires. Patients were asked to specify marital status (married/living with partner, separated, divorced, widowed, single, not specified), living status (alone, with others) and travel distance (up to 30 min, between 30 min and 1 h, more than 1 h) to the training center. For a baseline assessment of physical activity reflecting the last week prior to inclusion we used the short version of the Freiburg questionnaire of physical activity (FFKA) [20], a standardized and validated instrument. FFKA focuses on everyday activity with low to medium exertion in addition to exercise activities. With 8 questions basal, recreational and sportive activities are assessed. Cancer-related fatigue, the primary endpoint of the BEST-study, was measured by the multidimensional, 20-item Fatigue Assessment Questionnaire (FAQ) [21]. FAQ is a standardized and validated questionnaire for self-assessment of the physical, cognitive and affective dimensions of fatigue. In addition, using a visual analogue scale (0 = not tired at all, 10 = totally exhausted), patients were asked to rate the intensity of current fatigue. Besides the continuous fatigue scores (total physical, cognitive and affective fatigue) we also investigated a dichotomous variable using as cutpoint the mean fatigue score of women age ≥ 60 years from the general German population (FAQ point value for women aged 14–39 years: 11,2; aged 40–59 years: 12,1; aged ≥ 60 years) [22].To assess the motivation of patients to participate in the BEST-study they could choose among 7 answers, in addition to provide their own free text. Nonparticipants had the choice between 15 answers and free text to explain why they decided against study participation. Multiple answers were allowed.

If the questionnaires were not returned, patients were reminded at least three times by phone. All questionnaires and clinical information had to be completed prior to the first radiotherapy.

### Statistical analysis

Characteristics of participants and nonparticipants were compared using Student’s *t*-test (metric data), Mann–Whitney-*U*-test (ordinal data), or Chi^2^-test (categorical data). To assess the associations between the characteristics and nonparticipation, we performed multiple logistic regression analyses assessing following covariates simultaneously in the model: age group, number of comorbidities, marital status, living status, travel distance, BMI, previous chemotherapy, and fatigue. Due to high collinearity tumor characteristics were investigated in separate models without chemotherapy. The different physical activity variables were also included in the models, but none showed a significant association with nonparticipation. Additional sensitivity analyses were performed using smaller sets of covariates to check for overfitting or spurious effects by related variables, but there were no substantial changes in the results. Reasons for participation or declination were analyzed descriptively. P-values below 0.05 were considered as statistically significant. All analyses were performed using PASW Statistics 19.0 or SAS 9.2 [SAS Institute Inc., Cary, USA].

## Results

From July to December 2011 a total of 398 patients with non-metastatic breast cancer were irradiated at the study clinic. In this period 52 participants and 69 nonparticipants were recruited (Figure 1). Due to missing data from 4 nonparticipants the sample was restricted to 65 evaluable nonparticipants. The remaining 277 patients were either not eligible, received no information from the recruiting physicians or were not interested in any study participation. Thus, information for this BEST-Participation-study was available for 30.4% of all breast cancer patients with adjuvant radiotherapy during the study period.

### Characteristics of participants and non-participants

Sociodemographic characteristics are presented in Table 1. The mean age did not differ statistically significantly between participants and nonparticipants (p = 0.64). However, participants were more likely to be middle aged (age 40–70 years) whereas nonparticipants were more likely to be younger or older (<40 years, ≥ 70 years) (p = 0.02). Significant differences were also noted for living status (p = 0.009) and travel distance (p = 0.02). Here, the nonparticipants more commonly reported living alone and had to spend more time to travel from their home to the treatment center. There were no significant differences with respect to marital status (p = 0.18).

**Table 1:** **Sociodemographic and clinical characteristics of participants and nonparticipants**

	**Total (n = 117)**	**Participants (n = 52)**	**Nonparticipants (n = 65)**	**p-Value**
**Mean age (SD)**	57.0 (11.3)	56.5 (9.3)	57.5 (12.7)	0.64
**Age groups**				0.02
<40 years	7 (6%)	1 (1.9%)	6 (9.2%)	
40-49 years	24 (20.5%)	14 (26.9% )	10 (15.4%)	
50-59 years	37 (31.6%)	17 (32.7%)	20 (30.8%)	
60-69 years	31 (26.5%)	17 (32.7%)	14 (21.5%)	
≥70 years	18 (15.4%)	3 (5.8%)	15 (23.1%)	
**Marital status**				0.18
Married/living with partner	78 (66.7%)	38 (73.1%)	40 (61.5%)	
Separated	3 (2.6%)	2 (3.8%)	1 (1.5%)	
Divorced	11 (9.4%)	6 (11.5%)	5 (7.7%)	
Widowed	18 (15.4%)	5 (9.6%)	13 (20%)	
Single	7 (6.0%)	1 (1.9%)	6 (9.2%)	
**Living status**				0.01
Alone	32 (27.4%)	8 (15.4% )	24 (36.9%)	
With others	85 (72.6%)	44 (84.6%)	41 (63.1%)	
**Travel distance**				0.02
Up to 30 minutes	30 (25.6% )	18 (34.6%)	12 (18,5%)	
Between 30 minutes and 1 h	67 (57.3%)	30 (57.7%)	37 (56.9%)	
More than 1 h	20 (17.1%)	4 (7.7%)	16 (24.6%)	
**Mean BMI [kg/m** ^**2**^ **] (SD)**	26.3 (4.5)	25.7 (4.7)	26.9 (4.4)	0.14
**Tumor size**				0.047
Tis	16 (13.7%)	8 (15.4%)	8 (12.3%)	
T1	66 (56.4%)	34 (65.4%)	32 (49.2%)	
T2	30 (25.6%)	9 (17.3%)	21 (32.3%)	
T3	5 (4.3%)	1 (1.9%)	4 (6.2%)	
**Nodal status**				0.04
Positive	34 (29.1%)	10 (19.2%)	24 (36.9%)	
Negative	83 (70.9%)	42 (80.8%)	41 (63.1%)	
**Tumor stage (UICC)**				0.04
0	15 (12.8%)	8 (15.4% )	7 (10.8%)	
1	55 (47.0%)	29 (55.8%)	26 (40.0%)	
2	35 (29.9%)	11 (21.2%)	24 (36.9%)	
3	12 (10.3%)	4 (7.7%)	8 (12.3%)	
**Number of comorbidities**				0.07
0	48 (41.0%)	25 (48.1%)	23 (35.4%)	
1	43 (36.8%)	20 (38.5%)	23 (35.4%)	
2	14 (12.0%)	3 (5.8%)	11 (16.9%)	
≥3	12 (10.3%)	4 (7.7%)	8 (12.3%)	
**Chemotherapy**				0.09
yes	46 (39.3%)	16 (30.8%)	30 (46.2%)	
no	71 (60.7%)	36 (69.2%)	35 (53.8%)	
**Fatigue**				
any	83 (70.9%)	43 (82.7%)	40 (61.5%)	0.01
none	34 (29.1%)	9 (17.3%)	25 (38.5%)	

SD: Standard Deviation.

Tumor size (p = 0.047), nodal status (p = 0.036) and tumor stage (p = 0.043) were indicative for a locally more advanced disease among nonparticipants. The number of comorbidities tended to be higher in nonparticipants (p = 0.066). Prior treatment with chemotherapy was somewhat more frequently reported by nonparticipants (p = 0.09). No difference between both groups was observed for BMI (p = 0.14).

Participants and nonparticipants had comparable current physical activity levels (p > .99) (see Table 2). Only 34.6% of the participants and 33.8% of the nonparticipants were classified as “sufficiently active” according to the criteria of Frey and Berg [23] (total score in the FFKA of 30 or a score of 14 in sportive activities) prior to starting radiotherapy. Compared to a healthy reference group [20] our entire study population was comparably active in day-to-day and recreational activities, but less so with respect to exercise activities.

**Table 2:** **Physical activity levels of participants and nonparticipants (BEST-study) according to the short version of the FFKA**

	**Total (n = 117)**	**Participants (n = 52)**	**Nonparticipants (n = 65)**	**p-Value**
Total activity score* (SD)	27.9 (25.8)	28.2 (26.4)	27.6 (25.5)	>0.99
Basal activities* (SD)	10.6 (11.4)	10.6 (11.3)	10.6 (11.6)	0.79
Recreational activities* (SD)	12.5 (12.4)	13.0 (12.1)	12.1 (12.8)	0.48
Sportive activities* (SD)	4.8 (11.9)	4.6 (13.2)	4.9 (10.8)	0.89

*MET x hours/week.

SD: Standard Deviation, MET: metabolic equivalent of task.

Neither total fatigue levels (p = 0.25) nor levels for the subcategories physical, cognitive, and affective fatigue or sleep problems differed significantly between the groups (see Table 3). There was also no significant difference between participants and nonparticipants with regard to their fatigue levels the year before diagnosis, at the days after surgery, or during chemotherapy (if applicable). However, among nonparticipants significantly (p = 0.01) more women were currently free of fatigue (i.e. with levels below the mean fatigue level of the general reference population) than among participants.

**Table 3:** **Fatigue levels of participants and nonparticipants (BEST-study) according to FAQ**

	**Total (n = 117)**	**Participants (n = 52)**	**Nonparticipants (n = 65)**	**p-Value**
**Total fatigue (SD)**	22.1 (13.8)	23.5 (11.1)	20.9 (15.7)	0.25
**Physical fatigue (SD)**	12.4 (8.9)	12.8 (7.7)	12.1 (9.8)	0.43
**Cognitive fatigue (SD)**	2.9 (2.6)	3.2 (2.2)	2.7 (2.8)	0.14
**Affective fatigue (SD)**	5.4 (4.0)	6.1 (3.7)	4.9 (4.2)	0.06
**Sleep problems**	1.4 (1.1)	1.5 (1.0)	1.3 (1.1)	0.24

SD: Standard Deviation.

Multiple logistic regression analyses (see Table 4) showed significantly higher odds for nonparticipation among patients with higher age, higher number of comorbidities, those living alone, having longer travel distances, having received a chemotherapy, or those not feeling fatigued. Divorced or separated women had lower odds to refuse participation. BMI and physical activity were not associated with participation.

**Table 4:** **Multiple logistic regression model assessing the odds for nonparticipation**

**Covariates**	**OR (95% CI)**	**p**
**Age groups**		**0.025**
<40 years	6.5 (0.5 – 86.4))	
40-49 years	Ref.	
50-59 years	3.7 (0.9 – 15.8)	
60-69 years	1.0 (0.2 – 5.7)	
≥70 years	**16.1 (1.7 – 150.4)**	
**Number of comorbidities**		**0.031**
0	Ref.	
1	2.8 (0.8 – 10.0)	
≥2	**10.6 (1.8 – 62.1)**	
**Marital status**		**0.047**
Married/ living with partner	Ref.	
Separated/divorced	**0.1 (0.02 – 0.9)**	
Widowed	0.2 (0.03 – 1.3)	
Single	14.3 (0.4 – 581.8)	
**Living status**		**0.039**
With others	Ref.	
Alone	**6.5 (1.1 – 38.3)**	
**Travel distance**		**0.0012**
Up to 30 minutes	Ref.	
Between 30 minutes and 1 h	**12.3 (2.7 – 56.7)**	
More than 1 h	**19.4 (3.4 – 110.8)**	
**Body mass index**		0.33
Normal	Ref.	
Overweight	2.2 (0.7 – 7.0)	
Adipose	2.3 (0.5 – 10.1)	
**Chemotherapy**		**0.0066**
No	Ref.	
Yes	**5.3 (1.6 – 17.8)**	
**Fatigue**		**0.0007**
Any	Ref.	
None	**12.7 (2.9 – 54.9)**	

OR: odds ratio; CI: confidence interval.

Bold numbers indicate statistically significant results at level 0.05.

Due to high collinearity, the variables tumor stage, tumor size, and chemotherapy were not included jointly in a model. Similar results were observed in the models with either tumor stage or tumor size instead of chemotherapy, with OR = 3.3 (1.2 – 9.3) for higher stage vs. lower stage, and OR = 4.6 (1.4 – 15.1) for tumor sizes T2/3 vs. T1/Tis (data not shown).

### Reasons for participation or non-participation

When asked for their reasons for acceptance or declination of the study the five most frequently stated motivational aspects of BEST participants were: “I hope to reduce fatigue and improve quality of life” (65.4%), “I want to contribute to scientific progress” (65.4%), “I hope for positive effects on my cancer prognosis” (59.6%), “I want to cope with cancer” (59.6%), “I hope for better fitness and health” (57.7%) (see Table 5). Nonparticipants most often named “Journey too far/difficulties to reach training center” (70.8%) as reason for their declination. “Time conflicts” (23.1%) and “Twice a week training is too much” (13.8%) were followed by “Health problems” and “I do not want to participate in relaxation” (both 12.3%) (see Table 6).

**Table 5:** **Motivations for study participation reported by participants of the BEST-study**

**Participant’s motivations (n = 52)**	
I want to contribute to scientific progress, that future patients benefit thereof	65.4%
I hope to reduce fatigue and improve quality of life	65.4%
I hope for positive effects on cancer prognosis	59.6%
I want to cope with cancer	59.6%
I hope for better fitness and health	57.7%
Social contact to other persons concerned	38.5%
Recommendation of the physician	28.8%
Other reasons	7.7%

**Table 6:** **Reasons for declination of study participation given by nonparticipants of the BEST-study**

**Nonparticipant’s reasons (n = 65)**	
Journey too far/difficulties to reach training centre	70.8%
Time conflicts	23.1%
Twice a week training is too much	13.8%
Health problems	12.3%
I do not want to participate in relaxation	12.3%
Dislike of randomization	10.8%
Additional time for questionnaires and measurements is too much	9.2%
Pain	9.2%
Resistance training too exhausting	7.7%
Too tired or not motivated enough	6.2%
I can’t be bothered	3.1%
Doubts about meaningfulness of the study	1.5%
Doubts about benefit of the training	1.5%
Investigation too burdensome/ inconvenient	1.5%
1 h training/session is too much	1.5%
Prefer not to give reasons	1.5%
Other reasons	15.4%

## Discussion

The present study showed that willingness of breast cancer patients to participate in a randomized exercise intervention study was significantly influenced by age distribution, comorbidities, living status, travel time to the training center, cancer characteristics or treatment, and fatigue. No associations were observed with BMI and extent of current physical activity.

Patient’s well-being often is negatively influenced by cancer itself as well as by common cancer therapies. Intervention studies have revealed the importance of physical activity as an effective method in supportive therapy [1,2,4,5].

In literature, generalizability of results from clinical trials to the overall patient population has been questioned [12,14,24]. Discrepancies between willingness in general expressed by healthy persons to participate in a clinical cancer trial versus actual participation rates in patient populations have been addressed in the study from Comis et al. 2003: In a survey healthy Americans were asked about their willingness to participate in a clinical cancer trial if they were faced with cancer diagnosis. 31% indicated to be “very willing”, 51% “somewhat willing”, 9% “not very willing” and 6% “not willing at all” [25]. This is in clear contrast to the finding that only 3 to 5% of all cancer patients in Great Britain and the US participate in clinical trials [9]. Reasons may be that no appropriate study was open, patients were not informed about ongoing studies or were ineligible, or patients were not willing to participate. In the BEST-study 13.1% of the target population could be enrolled during the recruitment period (from July to December 2011). In consequence it is assumed that participation in intervention studies is highly selective.

In the present study, travel distance was a major factor reducing study participation: Participation in the intervention trial required the acceptance to travel twice a week to the treatment center and to spend extra time for the training. The present study shows that patients who needed significantly less time to reach the training centre were more likely to agree. Furthermore, 70.8% of the nonparticipants named “journey too far/difficulties to reach training centre” as an important reason for refusing to participate. Long travel distances and limited transportation capabilities are held responsible for less utilization of cancer treatment [26]. In line, studies from gynecological cancer types reported that travel distance and study participation are reciprocally related to each other [15].

Participants of the BEST-study represented the age group between 40 to 69 years very well. However, patients aged younger than 40 years or older than 69 years only accounted for 7.7% of the trial participants whereas 32.2% of the nonparticipants were classified in these groups. Higher age was even significantly associated with nonparticipation when adjusting for other factors such as comorbidities. Our results strengthen previous studies observing that especially older patients are underrepresented in clinical trials, although they comprise about one third of newly diagnosed breast cancer patients [12,27,28]. A detailed explanation for the selected representation of younger patients cannot be determined due to the small size of this age group.

In the BEST-Participation-study among nonparticipants a larger proportion reported living alone. Participants tended to be more often married, while widowed or single women were more frequent among nonparticipants, but differences were not statistically significant. Our data suggest that social and perhaps logistical support is an important component when considering participation in a rather time-consuming clinical exercise trial. These data are in line with Elting et al. [12] who compared social factors of participants and nonparticipants with different cancer types. However, two other studies could not find such associations [17,29].

Comparable to Elting et al. who observed higher percentages of comorbidities in nonparticipants [12], the present study revealed a statistical association between more comorbidities and nonparticipation when adjusting for other characteristics. Participants had smaller tumor sizes, lower tumor stages and less frequently positive lymph nodes than nonparticipants. Potentially, physical activity intervention trials are selective for patients with less advanced and rather local disease.

Importantly, nonparticipants and participants did not differ in their physical activity level and both groups showed a predominantly sedentary lifestyle. Thus, the present study suggests that exercise intervention studies are not selectively addressing more active patients. Likewise the BEST-Participation-study found no influence of BMI on participation in an exercise trial. As the BEST-study focuses on interventions against fatigue, it is not surprising that patients who did not feel fatigued at all were more likely to refuse study participation.

In the literature two main theories are discussed to explain motivation of participants to participate in clinical trials: Altruistic motives as well as self-interested arguments. Jenkins and Fallowfield [30] found that a major argument for participation in clinical trials is the desire to help future patients who are confronted with cancer diagnosis. In contrast to these results Truong et al. [31] showed that altruism is not the primary reason for accepting participation. Catt et al. [32] also discussed personal benefits to be responsible for acceptance or non-acceptance of trial inclusion. In the Best-Participation-study we observed both of these motivations. “I hope to reduce fatigue and improve quality of life” and “I want to contribute to scientific progress, that future patients benefit thereof” were the two most frequent statements. Thus, the feeling to influence cancer prognosis and to positively impact the own health status seems both to be important to patients.

Declination of study participation in the BEST-study was largely attributable to the distance to get to the training centre or to difficulties related to commuting. Previous studies also showed that distance and limited transportation possibilities can be barriers for study participation [33,34]. In a review Ross et al. [35] concluded that long travel distances associated with higher costs led to a declination of study participation and higher dropout rates. “Date difficulties”, “Twice a week training is too much” and “Additional time for questionnaires and measurements is too much” can be summarized as additional logistical constraints that may be important in line with findings from other studies [34,36].

Our study had several strengths: By including only patients who were classified to be eligible through the BEST-study protocol and were offered participation from physicians we were able to investigate patient-related factors in relation to participation and nonparticipation. In contrast, most of the previous studies investigating these differences used retrospective designs where recruitment procedures and criteria were not fully comparable between participants and nonparticipants. Furthermore we collected additional information like fatigue status or physical activity level which are important variables in the emerging field of exercise interventions among cancer patients. A limitation of our BEST-Participation-study is, that as most of the clinical studies, we also cannot report on the exact percentage of contacted patients with respect to the entire target population. Due to organizational difficulties in the recruitment process, such as short-term alterations of the medical staff or time constraints of the physicians, not all patients were informed about the BEST-study, and among patients not willing to participate in BEST, not all were asked to complete the questionnaires of the BEST-Participation-study. Beyond that a certain proportion of the target population decided against any study participation, so that only 30.4% of all patients with breast cancer stage I-III responded to the BEST-Participation-study, either with participation or non-participation in the BEST-study. Further research is necessary to verify these aspects in detail.

## Conclusions

Our study suggest that breast cancer patients are less willing or able to participate in a randomized resistance exercise trial if they live alone, have a long travel distance, a worse cancer prognosis, had a recent chemotherapy, are affected by fatigue, are above age 70 or have more comorbidities. However, there seemed to be no selection by current physical activity level or BMI. By far the most frequently reported reason for declination of participation was too long commuting time to the training facility. Our results might help to improve participation by adapting recruitment procedures in future exercise intervention studies appropriately.
